# The general practitioner in charge of addictive behavior

**DOI:** 10.1192/j.eurpsy.2023.1418

**Published:** 2023-07-19

**Authors:** W. Bouali, R. Omezzine Gniwa, N. Faouel, R. Ben Soussia, L. Zarrouk

**Affiliations:** 1 Psychiatrie, Faculty of Medicine of Monastir, Mahdia; 2 family medicine, Faculty of Medicine of Monastir, Monastir, Tunisia

## Abstract

**Introduction:**

Recent reports confirmed that more than 22% of adult world population are suffering from addiction. Tobacco and alcohol use remain the most prevalent addictive behaviors reported in Tunisia. The management of addiction is a multidisciplinary team concept. This entity may be underdiagnosed due to perception default at the first line of management.

The aim of this study was to identify the limiting factors for addictive behavior approach in general practitioner (GP) clinic.

Recent reports confirmed that more than 22% of adult world population are suffering from addiction. Tobacco and alcohol use remain the most prevalent addictive behaviors reported in Tunisia. The management of addiction is a multidisciplinary team concept. This entity may be underdiagnosed due to perception default at the first line of management.

**Objectives:**

The aim of this study was to identify the limiting factors for addictive behavior approach in general practitioner (GP) clinic.

**Methods:**

A cross-sectional study involved 84 GPs in the city of Monastir, Tunisia. Self‐reported questionnaire was designed to survey the prevalence of patients with detectable addictive behavior among the outpatient GP clinic visitors.

**Results:**

The participation rate was 93.3% (84/90). The prevalence of addictive behaviors was variable (38-59.5%). Tobacco use was the most common addiction (91.7%). More than seventy percent of questioned GPs were regularly consulting patients with known addictive behavior. The diagnosis was incidental in 7% of cases. Sixty percent of patients had predisposing factors for addiction. Diagnostic with screening difficulties for addictive behavior were independently related to doctor’s age >40 (OR = 6.51; p = 0.005), exercise in private clinic (OR= 6.46; p=0.004). Thirty-three percent of GPs were more involved in addiction monitoring. The use of assessment scales was noted in 15%. Young physician age (OR=5.20; p=0.002) and the absence postgraduate diploma in addictology (OR=9.66; p=0.01) were significantly associated addiction management avoidance.

**Conclusions:**

This study aimed to assess of the attitude of GP in Monastir city regarding the addictive behaviors of their patients. The diagnosis and the management of addiction is not standardized for these health practitioners and this will not contribute to the battle against this social entity.

**Disclosure of Interest:**

None Declared

